# Coordinating source-sink dynamics and canopy photosynthesis through organic fertilizer substitution synergistically enhances foxtail millet yield and quality

**DOI:** 10.3389/fpls.2026.1938408

**Published:** 2026-08-24

**Authors:** Changhong Liu, Yizhou Liu, Ling Qin, Yanbing Yang, Feifei Li, Huawen Zhang, Hailian Wang, Runfeng Wang, Yingxing Zhao, Bing Liu, Erying Chen, Yanan Guan

**Affiliations:** Featured Crops Engineering Laboratory of Shandong Province, Crop Research Institute, Shandong Academy of Agricultural Sciences, Jinan, China

**Keywords:** foxtail millet, nitrogen fertilizer reduction, organic fertilizer substitution, sink and source relationships, beta function model

## Abstract

Replacing chemical nitrogen (N) fertilizer with organic fertilizer is widely considered an effective approach to sustain and enhance crop yields under reduced chemical fertilizer inputs. However, the underlying physiological mechanisms remain unclear. This study quantified source-sink growth dynamics and canopy photosynthetic traits in foxtail millet (*Setaria italica* L.) under varying N reduction ratios (15%, N-15; 30%, N-30) and organic substitution ratios (15%, N+15org; 30%, N+30org) relative to conventional N application (CT, 150 kg ha^-1^). The results indicated that moderate N fertilizer reduction (15%) exerted no significant adverse effects on grain yield or quality. However, compared with the CT treatment, the N-30 treatment significantly decreased panicle weight and total grain yield by 5.6—8.9% and 9.5—11.2%, the contents of yellow pigment, starch, and protein were lowered by 15.4—30.1%, 2.7—3.6%, and 5.7—9.2%, source and sink activities were significantly suppressed by 21.9—27.3% and 6.1—14.1%, respectively (*p* < 0.05). Conversely, replacing 30% of chemical N with organic fertilizer (N+30org) significantly increased grain yield by 17.3—23.5%, grain protein content by 6.6—12.2%, and the amylopectin/amylose ratio by 7.5—17.9% relative to CT (*p* < 0.05). The N+30org treatment enhanced the leaf area index (LAI, 7.0—11.3%), net photosynthetic rate (Pn, 10.6—21.2%), transpiration rate (Tr, 12.1—24.4%), stomatal conductance (Gs, 9.6—16.4%), and the actual quantum efficiency of PSII (ΦPSII, 13.2—15.7%) during the late grain-filling stage (30—40 days after anthesis). Concurrently, the time required to reach peak sink activity (t_m,o_) was prolonged by 6—11 days by the organic substitution strategy, which also boosted both source and sink activities, expanded the maximum sink biomass (W_max,h_) by 9.8—15.7%, and increased the sink-to-source ratio by 8.7—11.4%. Random Forest importance assessment and partial least squares path modeling (PLS-PM) revealed that grain yield formation was closely associated with sink and source relationships, whereas canopy photosynthetic characteristics may contribute to grain quality. Substituting 30% of chemical N with organic fertilizer was associated with a slower decline in canopy photosynthetic performance and mitigated source supply limitations induced by N fertilizer reduction. Consequently, high photosynthetic capacity during the late grain-filling stage was maintained, and source-sink capacity and activity were substantially enhanced, thereby achieving a synergistic optimization of high yield and superior quality in foxtail millet.

## 1 Introduction

Foxtail millet (*Setaria italica* L.) is widely cultivated as a vital food security crop in global arid and semi-arid regions due to its prominent drought-resistant and barren-tolerant adaptability (El-Beltagi et al., 2025). Grain of foxtail millet is richly endowed with essential amino acids required by the human body (e.g., leucine, isoleucine, and phenylalanine), a high proportion of dietary fiber, unsaturated fatty acids, as well as diverse vitamins and trace mineral elements (Zhao et al., 2026). Aligned with the international paradigm of dietary diversification, immense application potential and health benefits are held by foxtail millet in the development of modern diversified dietary structures and functional foods (Anwar et al., 2026). An important role is played by nitrogen (N) fertilizer in regulating crop yield and nutrient metabolic pathways, photosynthesis and dry matter accumulation in foxtail millet are determined by different N supply levels, reasonable N fertilizer input can increase grain protein and starch content, regulate the ratio of amylopectin to amylose, and improve appearance and eating quality (Mao et al., 2024). However, driven by the pursuit of maximizing crop yields, excessive chemical N application has become a pervasive phenomenon in global agriculture. Massive quantities of applied N cannot be efficiently assimilated by crops, resulting in substantial losses via ammonia volatilization, nitrous oxide emissions, and nitrate leaching, the global greenhouse effect, soil acidification, and aquatic eutrophication have been severely exacerbated (Palmero et al., 2026). Currently, chemical fertilizer reduction has been accepted as a core consensus in global agricultural management policies, however, how a “win-win” scenario between chemical fertilizer reduction and food security can be achieved remains an urgent problem to be resolved in sustainable agricultural development.

Various agronomic compensation and nutrient management practices have been explored in contemporary agricultural scientific research to effectively mitigate the potential risk of crop yield penalties induced by chemical fertilizer reduction. The negative impacts of chemical fertilizer reduction on crop yields can be alleviated to a certain extent by fertilizer management practices, including the application of controlled-release urea (Ding et al., 2024), organic fertilizer substitution (Nie et al., 2025), deep fertilization placement (Hu et al., 2023), and green manure cultivation (Kumar and Bordoloi, 2024), as demonstrated in previous studies. Nevertheless, feasibility analyses encompassing economic costs, environmental benefits, additional labor inputs, and technological constraints must be comprehensively evaluated before these agricultural practices can be widely promoted. Organic fertilizers produced via the composting and decomposition of agricultural wastes such as crop straw and livestock manure, which directly reduce the cost and increase the efficiency of farmers and drive the recycling of resources (Kolawole et al., 2025). Additionally, core advantages are possessed by organic fertilizers in terms of sustaining farmland soil health, activating nutrients, and protecting ecological environments. Substantial inputs of organic carbon and trace mineral elements are delivered to farmland soils by organic fertilizer application; concurrently, the distribution of water-stable soil aggregates is improved, and soil water-holding, moisture-conservation, and buffering-infiltration capacities are enhanced by macromolecular organic matter (Wu et al., 2024). Regarding soil microecology, abundant metabolic substrates are provided for rhizosphere microorganisms by organic fertilizers, whereby soil bacterial and fungal community structures are effectively altered, and microbial diversity as well as the complexity and stability of network topological structures are strengthened (Lin et al., 2025). The activities of soil carbon, nitrogen, and phosphorus acquiring enzymes (e.g., urease, invertase, alkaline phosphatase, and dehydrogenase) can also be stimulated by organic fertilizer application, allowing a higher soil quality index to be established within typical agroecosystems (Xu et al., 2023). Currently, existing research related to organic fertilizers remains heavily concentrated on the evolution of soil fertility, soil quality improvement, and microbial community remodeling, whereas direct logical evidence linking organic fertilizer application to crop yield remains relatively deficient, soil improvement is conventionally deemed a prerequisite for crop yield enhancement (Wang et al., 2025a). Consequently, a quantitative elucidation of the direct driving mechanisms of organic fertilizers on foxtail millet growth and yield formation remains lacking in current literature from the perspective of the dynamic balance of plant intrinsic physiology, particularly the “sink and source” relationship governing photosynthate allocation.

The sink and source relationships are the core intrinsic plant physiological mechanism of crop dry matter accumulation, carbon and nitrogen metabolism distribution, yield and quality formation (Rosado-Souza et al., 2023). Crop yield formation is essentially characterized as a dynamic evolutionary process in which photosynthates are continuously translocated from “source” organs to “sink” organs and transformed, the supply-demand matching degree and material conversion efficiency of internal carbon and N metabolism are reflected by the equilibrium relationship between source and sink (Ma et al., 2025). To capture the spatiotemporal evolution of crop source and sink capacities throughout the growing season, a sigmoidal beta (β) function growth model is commonly adopted in contemporary research to fit the biomass accumulation dynamics of vegetative and reproductive organs (Shi et al., 2017; Wang et al., 2025b). The traditional growth model has symmetry or fixed proportion restrictions, for example, the inflection point of the Logistic curve is strictly fixed at 50% of the maximum biomass, and the inflection point of the Gompertz curve is fixed at 1/e of the maximum value. In addition, compared with traditional growth models, the paramount advantage of the β function lies in the explicit calculation of dynamic parameters with definitive biological significance (Shao et al., 2021). For instance, the maximum dry matter weight of source or sink organs is represented by W_max,p_ or W_max,h_ (reflecting source capacity and total sink potential), the critical time node at which the daily dry matter accumulation rate of source and sink organs reaches its peak is denoted by t_m_ (the period of maximum source-sink activity), and the maximum daily dry matter growth rate of source and sink is reflected by S_max,p_ or S_max,h_ (characterizing the maximum source supply capacity or potential sink demand). This methodology has been extensively applied to major food crops, including potato, maize, wheat, and rice (Fang et al., 2024; Fei et al., 2024; Liao et al., 2024; Wang et al., 2025b). Crop yield potential is predominantly governed by the magnitude, activity, and duration of source and sink capacities, whereas grain quality attributes are determined not only by the transport of materials between source and sink but are also heavily dependent on leaf canopy photosynthetic traits (Ye et al., 2025). Therefore, a more comprehensive interpretation of the regulatory effects of organic fertilizer substitution on the yield and quality of foxtail millet can be achieved through a joint analysis combining canopy photosynthetic traits with sink and source relationships.

In summary, two N fertilizer reduction levels (15% and 30%) and their corresponding equivalent organic fertilizer substitution ratios (15% and 30%) were established in the present study. Dry matter dynamics and daily growth rates of foxtail millet source and sink organs were analyzed through a quantitative beta (β) function model, and the regulatory effects of different organic fertilizer substitution ratios on the yield and quality of foxtail millet were systematically evaluated in combination with canopy photosynthetic characteristics. We hypothesized that the organic fertilizer substitution can coordinate the source-sink balance and improve the canopy photosynthetic performance, thereby achieving the synergistic improvement of foxtail millet yield and quality under the condition of chemical fertilizer reduction. The objective of this study was to demonstrate that organic fertilizer substitution represents an effective fertilizer management practice for sustaining and enhancing foxtail millet production under chemical fertilizer reduction regimes.

## 2 Materials and methods

### 2.1 Experimental site

From May 2022 to October 2023, field experiments were conducted at the experimental field of the Jinan Experimental Station, Crop Research Institute, Shandong Academy of Agricultural Sciences (117°4’43.41”E, 36°42’24.01”N), which is located in a single-cropping region of spring foxtail millet within the North China Plain. The experimental site is situated in the transitional zone between the low mountains and hills of south-central and the alluvial plain of northwestern, with an elevation of 19 m. This region is characterized by a warm temperate continental monsoon climate, where the annual average temperature, annual sunshine duration, and annual average precipitation are 15.5°C, 2616.8 h, and 671.1 mm, respectively. Meteorological variations during the foxtail millet growing seasons are presented in **Supplementary Figure 1**. The soil type at the experimental site is classified as brown soil, which corresponds to Cambisols in the international soil classification system. Prior to the initiation of the experiment in 2022, the physicochemical properties of the topsoil layer (0–20 cm) were determined as follows: pH was 8.3, total nitrogen content was 0.89 g kg⁻¹, total phosphorus content was 0.62 g kg⁻¹, total potassium content was 13.1 g kg⁻¹, organic matter content was 14.1 g kg⁻¹, alkaline hydrolyzable nitrogen content was 55.9 mg kg⁻¹, and available phosphorus content was 13.4 mg kg⁻¹.

### 2.2 Experimental design

The newly high-yielding foxtail millet variety “Jigu 22”, developed by the Shandong Academy of Agricultural Sciences, was applied in the experimental, which is suitable for spring and summer sowing in northern China. Five distinct treatments were established: 1) conventional fertilization (control treatment, CT); 2) 15% N reduction (N-15); 3) 15% organic fertilizer substitution (N + 15org); 4) 30% N reduction (N-30); and 5) 30% organic fertilizer substitution (N + 30org). Equal nutrient inputs were strictly maintained across the CT, N + 15org, and N + 30org treatments, with application rates fixed at 150 kg ha⁻¹ of N (urea, N 46%), 67.5 kg ha⁻¹ of P_2_O_5_ (calcium superphosphate, P_2_O_5_ 16%), and 67.5 kg ha⁻¹ of K_2_O (potassium chloride, K_2_O 60%). For the N + 15org and N + 30org treatments, bio-organic fertilizer (Weifang Lvwangda Bio-fertilizer Technology Co., Ltd.) was applied at rates of 1500 kg ha⁻¹ and 3000 kg ha⁻¹, respectively. The organic fertilizer contained 1.5% N, 1.0% P, and 0.8% K; equivalent phosphorus and potassium balancing was achieved prior to fertilization by reducing the corresponding inputs of chemical phosphorus and potassium fertilizers based on the nutrient content of the organic fertilizer. All chemical fertilizers and organic fertilizers for each treatment were applied as basal fertilizers before sowing, with no topdressing supplied throughout the growing season. The fertilizers were evenly broadcast across each plot and thoroughly incorporated into the 0–20 cm topsoil layer using a rotary tiller during soil preparation. The area of each experimental plot was set to 20 m² (4 m × 5 m), with a row spacing of 0.5 m and a planting density maintained at 600,000 plants ha⁻¹. The experiment used a randomized block design and ensured that each plot was repeated three times. No irrigation was supplied throughout the entire growing season, and other field management practices for pests, diseases, and weeds were implemented in accordance with the technical guidelines for high-standard farmland foxtail millet production.

### 2.3 Measurements of foxtail millet biomass and yield

At 10, 20, 30, 40, 50, 70, 90, and 110 days after sowing, the aboveground biomass was sampled from 10 foxtail millet plants in each plot. The sampled plants were oven-dried at 105°C for 30 min for enzymatic inactivation, and then dried at 80°C to a constant weight before being weighed to calculate dry matter accumulation. At the maturity stage, 30 foxtail millet plants were randomly selected from each plot to determine plant height, stem diameter, panicle length, panicle diameter, and panicle weight. After threshing, the 1000-grain weight was determined. Concurrently, the grains were milled into dehulled grains using a laboratory rice huller (LTJM-2099, Shanghai, China) to calculate the milling percentage. For final grain yield determination, a 6 m² area from the central rows of each plot was harvested, air-dried, and threshed. The grain moisture content was determined using a grain moisture tester (PM-8188, Japan), and the final grain yield was converted based on a standard moisture content. Three replicates were performed for each treatment.

### 2.4 Foxtail millet quality

At the maturity stage of foxtail millet, 10 panicles were collected from each plot, grain quality attributes were subsequently evaluated after these panicles were air-dried, threshed, and dehulled. The protein content in foxtail millet grains was determined via the Kjeldahl method (Bogucka and Elżbieta, 2018). The total starch content was measured using the anthrone colorimetric method, whereas the amylose and amylopectin contents were quantified using the iodine colorimetric method (Haldar et al., 2017). The determination of fat content was performed through the Soxhlet extraction method (Mao et al., 2024). For the measurement of yellow pigment content, a standard curve was constructed using β-carotene; extraction was achieved using water-saturated n-butanol under shaking for 3 h, and the supernatant obtained after centrifugation was measured colorimetrically at 445 nm (Yang et al., 2017).

### 2.5 Photosynthetic parameters

The actual quantum efficiency of PSII (ΦPSII) and maximum ΦPSII quantum efficiency (Fv/Fm) were determined at 0, 10, 20, 30, and 40 days after anthesis using the FMS-2 Pulse Modulated Fluorometer (FMS-2, Hansatech, UK). A portable photosynthesis system (CIRAS-3, PP SYSTEMS, USA) was employed for the measurements of net photosynthetic rate (Pn), transpiration rate (Tr), intercellular CO_2_ concentration (Ci), and stomatal conductance (Gs). Concurrently, the relative chlorophyll content (SPAD) of foxtail millet flag leaves under each treatment was measured using a portable chlorophyll meter (SPAD-502, Konica Minolta, Japan), and the leaf area index (LAI) was determined using a canopy analyzer (LP-80, METER, USA). Three replicates were performed for each measurement indicator.

### 2.6 Quantification of sink and source relationships

With reference to the characterization methods of source-sink dynamics in crops such as rice, maize, wheat, and potato, the dynamic changes in the carbon source and carbon sink of foxtail millet were quantified based on the kinetic characteristics of crop biomass (Fang et al., 2024; Fei et al., 2024; Liao et al., 2024; Wang et al., 2025b). The beta (β) function developed by Yin et al. (2009) was adopted in the present study to accurately simulate the relationships between growth days and biomass accumulation in foxtail millet, the specific definitions of the relevant functional parameters are presented in **Supplementary Figure 2** and **Supplementary Table 1**. The biomass of vegetative organs (source, i.e., stems and leaves) and reproductive organs (sink, i.e., panicles) of foxtail millet are represented by W_plant_ and W_head_, respectively, which can be further employed to estimate the parameters of crop carbon sources and carbon sinks based on the observed biomass data of W_plant_ and W_head_. The functional Equations 1, 2 calculating the changes in vegetative organ biomass (W_plant_, g m^-2^) and reproductive organ biomass (W_head_, g m^-2^) over time are expressed as follows:

(1) $$W_{plant}=\{\begin{array}{cc} W_{\max,p}(1+\frac{t_{e,p}-t}{t_{e,p}-t_{m,p}}){\left(\frac{t}{t_{e,p}}\right)}^{\frac{t_{e,\text{p}}}{t_{e,p}-t_{m,p}}} & t\leq t_{e,p}\\ W_{\max,p} & t>t_{e,p} \end{array}$$

(2) $$W_{head}=\{\begin{array}{cc} W_{b,o}+(W_{\max,h}-W_{b,\text{h}})(1+\frac{t_{e,o}-t}{t_{e,o}-t_{m,o}}){\left(\frac{t-t_{b,o}}{t_{e,o}-t_{b,o}}\right)}^{\frac{t_{e,o}-t_{b,o}}{t_{e,o}-t_{m,o}}} & t_{b,o}\leq t\leq t_{e,o}\\ W_{\max,h} & t>t_{e,o} \end{array}$$

Where t represents the growth days (d); the biomass of vegetative and reproductive organs (g m^-2^) are denoted by W_plant_ and W_head_, respectively; the maximum biomass values of vegetative organs (source supply) and reproductive organs (sink demand) are represented by W_max,p_ and W_max,h_ (g m^-2^), respectively; the growth days when the growth rates of vegetative and reproductive organs reach zero are designated as t_e,p_ (d) and t_e,o_ (d), respectively; the growth days at which the maximum growth rates of W_plant_ and W_head_ are achieved are indicated by t_m,p_ (d) and t_m,o_ (d), respectively; the day of initial panicle appearance is denoted by t_b,o_ (d); and the initial panicle biomass at the time of initial appearance is represented by W_b,h_ (g m^-2^). Because the panicle biomass at initial appearance is approximately equal to zero, a default value of 0 g m^-2^ is assigned to the initial panicle biomass during the calculation process.

The source activity (S_p_, g m^-2^ d^-1^) and sink demand (S_h_, g m^-2^ d^-1^) of foxtail millet can be calculated through (Equations 3, 4), respectively:

(3) $$S_{\text{p}}=S_{\max,p}(\frac{t_{e,p}-t}{t_{e,p}-t_{m,p}}){\left(\frac{t}{t_{m,p}}\right)}^{\frac{t_{m,p}}{t_{e,p}-t_{m,p}}}$$

(4) $$S_{\text{h}}=S_{\max,\text{h}}(\frac{t_{e,o}-t}{t_{e,o}-t_{m,o}}){\left(\frac{t-t_{b,o}}{t_{m,o}-t_{b,o}}\right)}^{\frac{t_{m,o}-t_{b,o}}{t_{e,o}-t_{m,o}}}$$

Where the source activity of foxtail millet (g m^-2^ d^-1^) is represented by S_p_; the maximum rate of source activity at the time point t_m,p_ is denoted by S_max,p_, and the maximum rate of sink demand at the time point t_m,o_ is indicated by S_max,h_. The maximum values of foxtail millet source activity and sink demand are represented by S_max,p_ and S_max,h_, respectively, and their calculation Equations 5, 6 are expressed as follows:

(5) $$S_{\max,\text{h}}=(W_{\max,\text{h}}-W_{b,\text{h}})[\frac{2{\text{t}}_{e,o}-t_{m,o}-t_{b,o}}{\left(t_{e,o}-t_{b,o})(t_{e,o}-t_{m,o}\right)}]{\left(\frac{t_{m,o}-t_{b,o}}{t_{e,o}-t_{b,o}}\right)}^{\frac{t_{m,o}-t_{b,o}}{t_{e,o}-t_{m,o}}}$$

(6) $$S_{\max,\text{p}}=W_{\max}{,p}_{[}\frac{2t_{e,p}-t_{m,p}}{t_{e,p}(t_{e,p}-t_{m,p})}]{\left(\frac{t_{e,p}}{t_{m,p}}\right)}^{\frac{t_{m,p}}{t_{e,p}-t_{m,p}}}$$

The average rate of foxtail millet source activity ( ${\bar{S}}_{p}$) is defined as the average growth rate of vegetative organs within the t_e,p_ time period, whereas the average rate of sink ( ${\bar{S}}_{h}$) is characterized as the average growth rate of reproductive organs within the time period between t_b,o_ and t_e,o_. Their calculation Equations 7, 8 are expressed as follows:

(7) $$\bar{S_{p}}=\frac{W_{\max,p}}{t_{e,p}}$$

(8) $$\bar{S_{h}}=\frac{W_{\max,h}}{t_{e,o}-t_{b,o}}$$

The source supply and sink demand of foxtail millet throughout the entire growth period are represented by W_max,p_ and W_max,h_, respectively; therefore, the calculation Equation 9 for the sink-to-source ratio (%) is expressed as follows:

(9) $$\mathrm{Sin}k/Source\;(\%)=\frac{\mathrm{Sin}k\;demand}{Source_{\sup}ply}=\frac{W_{\max,\text{h}}}{W_{\max,p}}\times 100\%$$

### 2.7 Statistical analysis

Raw data recording and statistics were performed using Microsoft Excel 2024. Two-way analysis of variance (ANOVA) was conducted using SPSS version 24 (SPSS Inc., Chicago, IL, USA) to calculate the interaction effects between year × chemical fertilizer reduction and year × organic fertilizer substitution among treatments. Significant differences among different treatments were evaluated using the least significant difference (LSD) test, with the significance level set at *p* < 0.05. Visual graphing and plotting of the experimental data were performed using Origin 2026 software (Origin Lab, Northampton, MA, USA). Data fitting, Random Forest importance assessment, and structural equation modeling analysis were calculated using the minpack.lm, randomForest, and seminr packages in R version 4.4.3.

## 3 Results

### 3.1 Foxtail millet yield and quality

Foxtail millet grain yield was moderately decreased by chemical N reduction (**Table 1**); specifically, a reduction of 1.0–5.7% (*p* > 0.05) in grain yield compared with the conventional N treatment (CT) was induced by a 15% reduction in N application rate (N-15). However, when a 30% reduction in N application rate was implemented (N-30), grain yield was significantly reduced by 9.5–11.2% (*p* < 0.05), which was driven by a significant decrease of 5.6–8.9% (*p* < 0.05) in panicle weight compared with the CT treatment. Under the condition of constant total amount of fertilizer, foxtail millet grain yield could be effectively enhanced by substituting a portion of chemical fertilizer with organic fertilizer (**Table 1**). Compared with the CT treatment, a slightly increase of 7.2–14.9% in grain yield was observed under the 15% equivalent organic fertilizer substitution treatment (N + 15org); furthermore, grain yield was significantly increased by 17.3–23.5% (*p* < 0.05) and panicle weight was significantly increased by 9.3–11.0% (*p* < 0.05) by the 30% equivalent organic fertilizer substitution treatment (N + 30org).

**Table 1:** Characteristics of foxtail millet yield and yield components under different fertilization treatments.

Year	Treatment	Plant height (cm)	Stem diameter (mm)	Panicle length (cm)	Panicle diameter (mm)	Single panicle weight (g)	1000-kernel weight (g)	Yield (kg ha^-1^)	Milling rate (%)
2022	CT	119.0 ± 2.5 a	6.1 ± 0.3 a	20.3 ± 1.5 ab	20.5 ± 1.9 ab	16.1 ± 0.7 bc	2.72 ± 0.02 a	5324.8 ± 241.8 b	80.9 ± 6.3 a
	N-15	107.3 ± 5.8 ab	5.8 ± 0.2 ab	19.6 ± 1.1 ab	20.1 ± 1.8 ab	15.7 ± 0.9 bc	2.70 ± 0.03 a	5272.1 ± 178.9 bc	80.4 ± 2.1 a
	N+15org	114.3 + 6.7 ab	6.0 ± 0.3 a	20.3 ± 1.7 a	20.9 ± 1.6 ab	16,6 ± 0.9 ab	2.72 ± 0.01 a	5771.1 ± 350.6 ab	79.3 ± 5.6 a
	N-30	106.6 ± 5.4 b	5.3 ± 0.5 b	17.7 ± 1.1 b	17.9 ± 1.2 b	15.2 ± 0.8 c	2.72 ± 0.03 a	4791.5 ± 192.6 c	80.1 ± 3.4 a
	N+30org	115.4 ± 9.7 ab	5.5 ± 0.4 ab	20.5 ± 1.8 a	23.1 ± 2.6 a	17.6 ± 0.6 a	2.71 ± 0.02 a	6247.0 ± 385.3 a	81.6 ± 2.6 a
2023	CT	117.6 ± 3.1 a	6.3 ± 0.5 a	19.1 ± 0.7 ab	17.2 ± 1.0 b	14.6 ± 0.8 bc	2.71 ± 0.02 a	4922.2 ± 262.7 c	80.6 ± 3.5 a
	N-15	110.3 ± 4.9 ab	6.1 ± 0.3 a	18.2 ± 0.8 ab	17.5 ± 0.8 b	14.3 ± 1.4 bc	2.71 ± 0.02 a	4640.8 ± 222.6 cd	80.8 ± 2.1 a
	N+15org	115.7 ± 3.8 a	6.4 ± 0.2 a	18.6 ± 0.8 ab	18.1 ± 0.3 ab	15.4 ± 0.7 ab	2.72 ± 0.02 a	5403.7 ± 195.7 b	80.7 ± 4.4 a
	N-30	105.1 ± 1.7 b	6.0 ± 0.3 a	17.7 ± 1.1 b	16.9 ± 0.9 b	13.3 ± 0.7 c	2.73 ± 0.03 a	4422.3 ± 165.9 d	80.5 ± 3.4 a
	N+30org	115.1 ± 10.7 ab	6.3 ± 0.3 a	19.3 ± 0.5 a	19.0 ± 0.4 a	16.2 ± 0.6 a	2.73 ± 0.01 a	6077.4 ± 280.5 a	80.7 ± 2.1 a
Year (Y)		Ns	13.1***	4.8*	17.7***	11.9**	Ns	Ns	Ns
Chemical fertilizer reduction (C)		14.4***	Ns	5.1*	Ns	Ns	Ns	9.1**	Ns
Organic fertilizer substitution (O)		Ns	Ns	Ns	Ns	7.2**	Ns	18.9***	Ns
Y × C		Ns	Ns	Ns	Ns	Ns	Ns	Ns	Ns
Y × O		Ns	Ns	Ns	Ns	Ns	Ns	Ns	Ns

Values are mean ± standard errors, different letters mean that the difference of the means between treatments was significant (LSD test, *P* < 0.05). *F* values under ANOVA analysis were provided. And Ns means no significant, *P < 0.05. **P < 0.01. ***P < 0.001.

A significant effect of chemical N reduction on the yellow pigment content of foxtail millet was observed (**Table 2**, *p* < 0.05). Compared with the conventional N treatment (CT), a slight reduction of 10.4–12.6% in yellow pigment content was induced by the 15% N reduction (N-15), whereas a significant decrease of 15.4–30.1% (*p* < 0.05) was caused by the 30% N reduction (N-30). Furthermore, total starch and protein contents were also significantly decreased by 2.7–3.6% and 5.7–9.2% (*p* < 0.05), respectively, under the N-30 treatment. Significant effects on the starch structure and protein content of foxtail millet were exerted by organic fertilizer substitution (**Table 2**, *p* < 0.05). Compared with the CT treatment, the amylopectin/amylose ratio and protein content were slightly increased by 2.7–2.9% and 4.2–5.1%, respectively, under the 15% equivalent organic fertilizer substitution treatment (N + 15org); meanwhile, the amylopectin/amylose ratio and protein content were significantly increased by 7.5–17.9% and 6.6–12.2% (*p* < 0.05), respectively, by the 30% organic fertilizer substitution treatment (N + 30org).

**Table 2:** Differences in foxtail millet quality characteristics under different fertilization treatments.

Year	Treatment	Yellow pigment	Amylose	Amylopectin	Starch content	Ratio of amylopectin to amylose	Protein content	Fat content
		(mg kg^-1^)	(%)	(%)	(%)	(%)	(%)	(%)
2022	CT	26.2 ± 2.3 a	14.2 ± 0.3 ab	52.4 ± 1.2 bc	66.6 ± 1.0 a	3.7 ± 0.1 bc	8.8 ± 0.3 bc	3.6 ± 0.4 a
	N-15	22.9 ± 1.3 b	14.9 ± 0.3 a	51.4 ± 0.8 bc	66.3 ± 1.1 ab	3.5 ± 0.04 c	8.6 ± 0.6 c	3.4 ± 0.2 a
	N+15org	24.9 ± 1.7 ab	13.9 ± 0.1 b	53.1 ± 0.5 ab	67.0 ± 0.5 a	3.8 ± 0.1 b	9.4 ± 0.3 ab	3.6 ± 0.1 a
	N-30	18.3 ± 1.9 c	14.2 ± 0.5 ab	50.7 ± 1.2 c	64.8 ± 0.8 b	3.6 ± 0.2 bc	8.3 ± 0.4 c	3.3 ± 0.2 a
	N+30org	26.0 ± 3.3 a	13.0 ± 0.6 c	54.8 ± 1.6 a	67.8 ± 1.1 a	4.2 ± 0.3 a	9.7 ± 0.2 a	3.7 ± 0.2 a
2023	CT	25.9 ± 2.6 ab	14.5 ± 0.3 b	51.4 ± 1.2 b	65.9 ± .1.0 ab	3.5 ± 0.1 b	8.7 ± 0.4 bc	3.5 ± 0.2 a
	N-15	23.2 ± 1.8 bc	15.9 ± 0.2 a	49.0 ± 0.6 c	64.9 ± 0.6 bc	3.1 ± 0.1 c	8.4 ± 0.6 cd	3.6 ± 0.2 a
	N+15org	24.5 ± 2.2 ab	14.3 ± 0.1 b	52.0 ± 0.5 ab	66.3 ± 0.5 ab	3.6 ± 0.0 b	9.2 ± 0.4 ab	3.5 ± 0.2 a
	N-30	21.9 ± 3.0 c	14.1 ± 0.5 b	49.4 ± 0.8 c	63.5 ± 1.3 c	3.5 ± 0.1 b	7.9 ± 0.5 d	3.5 ± 0.2 a
	N+30org	27.5 ± 1.6 a	13.5 ± 0.4 c	53.7 ± 1.6 a	67.2 ± 1.3 a	4.0 ± 0.2 a	9.6 ± 0.3 a	3.8 ± 0.2 a
Year (Y)		Ns	Ns	Ns	Ns	Ns	Ns	Ns
Chemical fertilizer reduction (C)		9.0**	Ns	Ns	Ns	Ns	Ns	Ns
Organic fertilizer substitution (O)		ns	19.5***	6.5*	Ns	15.2***	12.8***	Ns
Y × C		Ns	Ns	Ns	Ns	Ns	Ns	Ns
Y × O		Ns	Ns	Ns	Ns	Ns	Ns	Ns

Values are mean ± standard errors, different letters mean that the difference of the means between treatments was significant (LSD test, *P* < 0.05). *F* values under ANOVA analysis were provided. And Ns means no significant, *P < 0.05. **P < 0.01. ***P < 0.001.

### 3.2 Canopy characteristics

**Figure 1:**
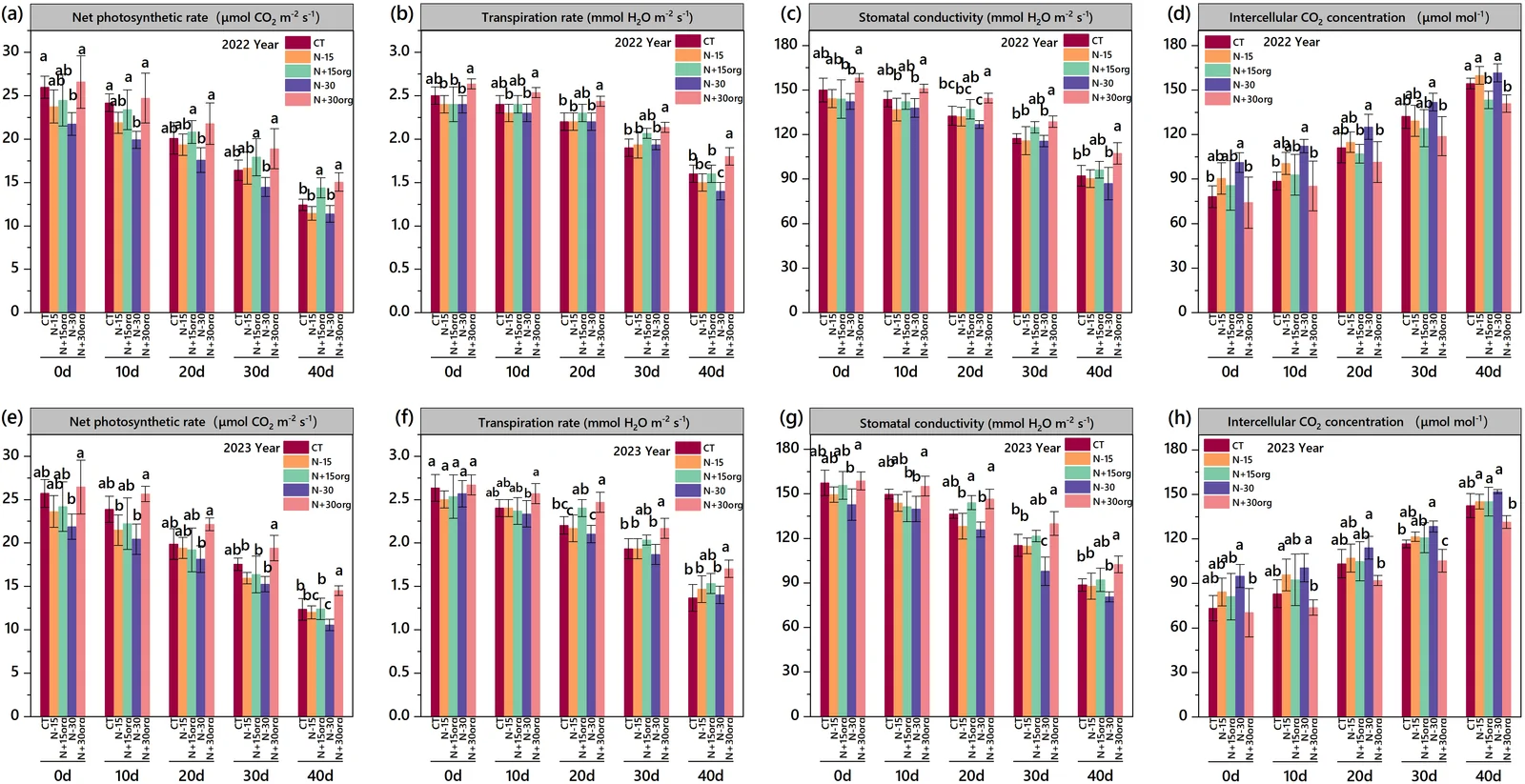
Grouped bar chart showing physiological parameters in two years, 2022 and 2023, across five treatments and five time points. Panels include net photosynthetic rate **(a, e)** (Pn, µmol CO_2_ m^-2^ s^-1^), transpiration rate **(b, f)** (Tr, mmol H_2_O m^-2^ s^-1^), stomatal conductance **(c, g)** (Gs, mmol H_2_O m^-2^ s^-1^) and intercellular carbon dioxide concentration **(d, h)** (Ci, µmol mol^-1^). Each panel compares red, green, brown, blue, and purple bars. Error bars and statistical annotation letters are present above each bar indicating group differences. For a detailed breakdown, the top row represents 2022 data and the bottom row represents 2023 data, with successive panels **(a–h)** representing the same parameter for each year.

The effects of different N treatments on the photosynthetic parameters (Pn, Tr, Gs, and Ci) of foxtail millet were significantly regulated by the growth stage (0–40 days after anthesis, DAA) (**Figure 1**). Gradual decreases in Pn, Tr, and Gs were observed from 30 DAA, whereas a corresponding increase in Ci was observed. The Pn, Tr, and Gs of foxtail millet throughout the entire grain-filling stage (0–40 DAA) were moderately decreased by N fertilizer reduction. Specifically, compared with the conventional N treatment (CT), a significant reduction of 8.3–17.7% (*p* < 0.05) in Pn was induced by the 30% N reduction (N-30) during 0–40 DAA. However, no significant difference in post-anthesis photosynthetic performance was detected between the 15% N reduction (N-15) and the CT treatment (*p* > 0.05). Effective enhancements in Pn, Tr, and Gs during the late grain-filling stage (30–40 DAA) were observed under partial chemical N fertilizer substitution with organic fertilizer (**Figure 1**). Compared with the CT treatment, Pn was significantly increased by 10.6–21.2%, Tr by 12.1–24.4%, and Gs by 9.6–16.4% (*p* < 0.05) during the late grain-filling stage (30–40 DAA) by the 30% equivalent organic fertilizer substitution treatment (N + 30org).

**Figure 2:**
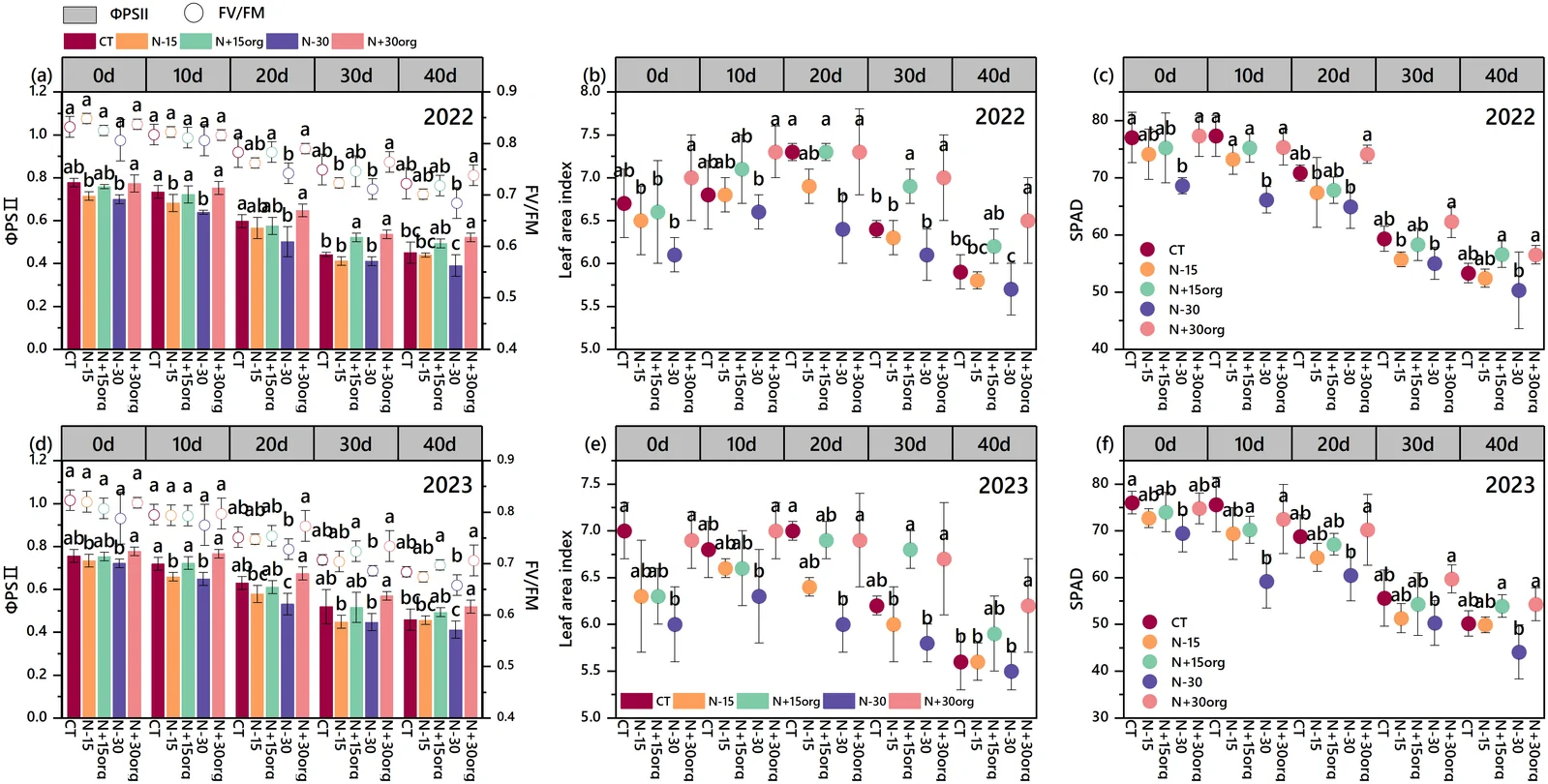
Grouped data visualization with six panels compares crop physiology measurements in 2022 and 2023 across five treatments over time. Bar and scatter plots display mean values and error bars for ΦPSII **(a, d)**, FV/FM **(a, d)**, leaf area index **(b, e)**, and SPAD **(c, f)** at intervals of 0, 10, 20, 30, and 40 days for CT, N-15, N+15org, N-30, and N+30org. Statistical significance is indicated by different letters above data points.

The actual photochemical efficiency (ΦPSII) and maximum photochemical efficiency (Fv/Fm) of foxtail millet during the grain-filling stage (0–40 DAA) were gradually decreased with the progression of the growth stage under different N treatments (**Figure 2**). No significant differences in ΦPSII and Fv/Fm within 40 DAA were observed between the chemical fertilizer reduction treatments (N-15 and N-30) and the CT treatment (*p* > 0.05). The highest ΦPSII and Fv/Fm values among all treatments were exhibited by the 30% organic fertilizer substitution treatment (N + 30org); particularly at the late grain-filling stage (40 DAA), a significant increase of 13.2–15.7% (*p* < 0.05) in ΦPSII compared with the CT treatment was driven by the N + 30org treatment.

Similar trends to the photosynthetic and fluorescence parameters were exhibited by the leaf area index (LAI) and relative chlorophyll content (SPAD) of foxtail millet during the grain-filling stage (0–40 DAA) (**Figure 2**). No significant effects on the LAI and SPAD values during the grain-filling stage were exerted by moderate reductions in the N application rate (*p* > 0.05). Conversely, the LAI during the late grain-filling stage (30–40 DAA) was effectively increased by partial chemical fertilizer substitution with organic fertilizer, wherein a significant increase of 7.0–11.3% (p < 0.05) in LAI during 30–40 DAA compared with the CT treatment was achieved under the N + 30org treatment.

### 3.3 Foxtail millet source and sink dynamics

**Figure 3:**
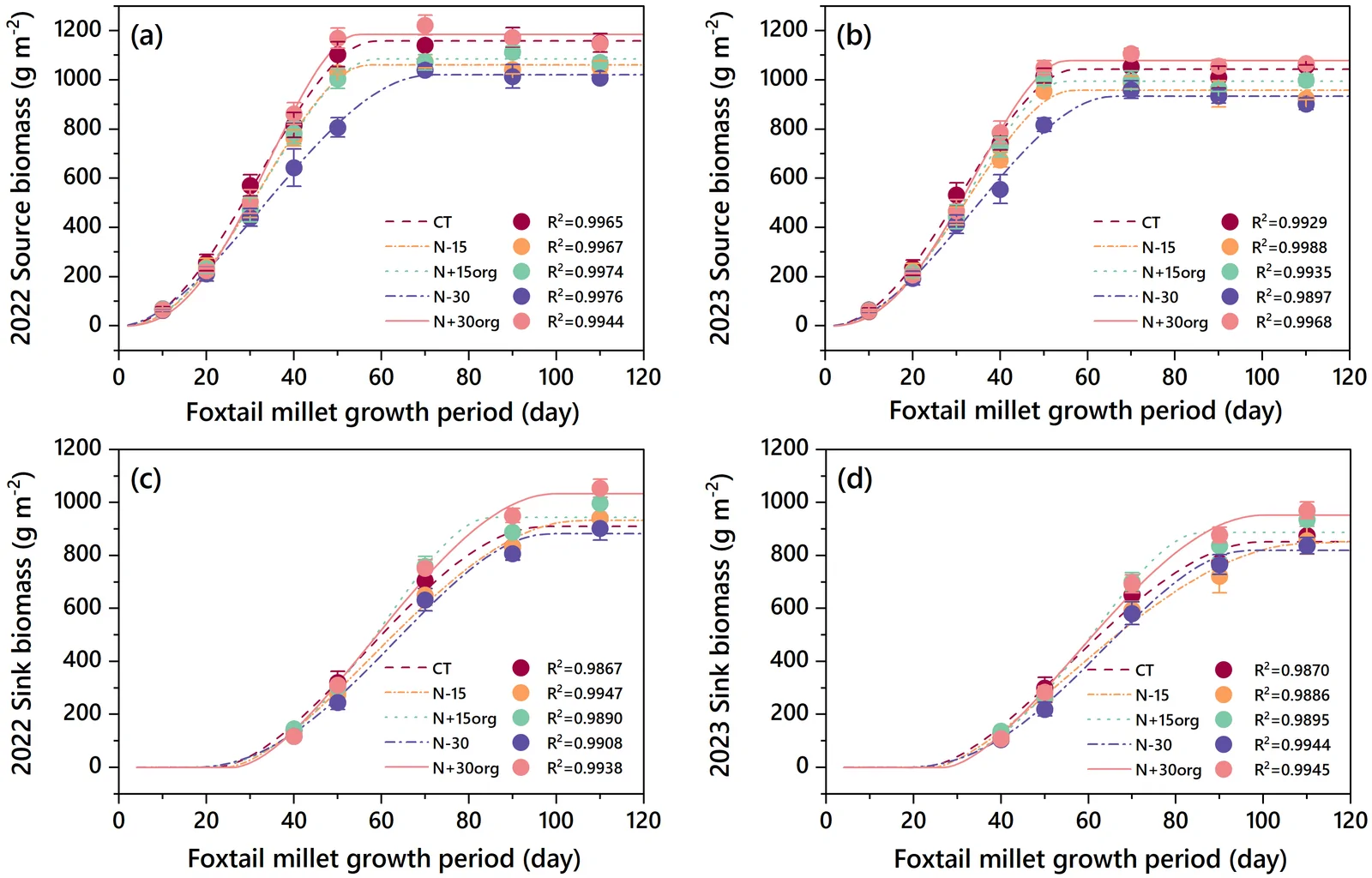
Histogram figure showing line graphs of foxtail millet growth period versus biomass. Panels **(a)** and **(b)** display source biomass for 2022 and 2023; **(c)** and **(d)** show sink biomass for 2022 and 2023. Each panel compares five fertilization treatments (CT, N-15, N+15org, N-30, N+30org) with R squared values represented by colored dots. Error bars mark data points.

The fitting results of dry matter data for foxtail millet source and sink using the beta (β) function (Equation 1) were presented in **Tables 3** and **4**, and **Figure 3**. High accuracy in simulating foxtail millet biomass across different growth periods was exhibited by the β function model, through which 98.7%–99.8% of the biomass variability was explained during the modeling process. The time required to reach peak foxtail millet source biomass (t_e,p_) was approximately 55–71 days, and the maximum source biomass (W_max,P_) ranged from 934.0 to 1185.9 g m^-2^. Concurrently, the initial time of foxtail millet sink biomass accumulation (t_b,o_) was approximately 14–27 days, the time required to reach peak sink biomass (t_e,o_) was approximately 86–113 days, and the maximum sink biomass (W_max,h_) ranged from 820.1 to 1033.9 g m^-2^.

**Table 3:** Parameter evaluation of foxtail millet source dynamics under different fertilization treatments.

Year	Treatment	Estimated parameter values of source					
		W_max,P_	t_e,p_	t_m,p_	S_max,p_	${\bar{S}}_{p}$	R^2^
		(g m^-2^)	(day)	(day)	(g m^-2^)	(g m^-2^ day^-1^)	
2022	CT	1158.4 ± 24.5 a	58.7 ± 3.3 b	31.9 ± 2.0 a	130.4	19.8 ± 0.9 b	0.9965
	N-15	1060.5 ± 21.7 bc	56.9 ± 1.2 bc	33.0 ± 1.6 a	133.7	18.6 ± 0.1 bc	0.9967
	N+15org	1085.7 ± 24.6 b	58.8 ± 1.1 b	33.4 ± 1.4 a	128.6	18.5 ± 0.8 c	0.9974
	N-30	1020.0 ± 25.1 c	71.0 ± 1.9 a	32.9 ± 2.4 a	79.9	14.4 ± 0.7 d	0.9976
	N+30org	1185.9 ± 20.4 a	54.9 ± 0.9 c	34.5 ± 1.2 a	175.5	21.6 ± 0.7 a	0.9944
2023	CT	1043.8 ± 17.3 a	57.4 ± 1.3 b	31.6 ± 1.7 a	122.0	18.2 ± 0.5 b	0.9929
	N-15	957.9 ± 26.6 bc	56.6 ± 1.1 bc	32.8 ± 1.5 a	120.9	16.9 ± 0.2 c	0.9888
	N+15org	995.1 ± 22.9 b	55.0 ± 0.7 c	33.5 ± 1.4 a	139.4	18.1 ± 0.5 b	0.9935
	N-30	934.0 ± 27.0 c	66.0 ± 1.0 a	34.0 ± 2.2 a	87.7	14.2 ± 0.5 d	0.9898
	N+30org	1079.6 ± 18.6 a	55.5 ± 0.8 c	34.3 ± 1.4 a	153.1	19.5 ± 0.6 a	0.9968
Year (Y)		19.0***	Ns	Ns	\	Ns	\
Chemical fertilizer reduction (C)		40.9***	78.5***	Ns	\	90.0***	\
Organic fertilizer substitution (O)		28.4***	4.7*	Ns	\	17.6***	\
Y × C		Ns	Ns	Ns	\	Ns	\
Y × O		Ns	Ns	Ns	\	Ns	\

Source parameter description: W_max,P_, the maximum source biomass; t_e,p_, timing when the rate of source supply is decreased to zero; t_m,p_, the moment when the maximum rate of source supply is achieved; S_max,p_, the maximum rate of source supply; ${\bar{S}}_{p}$, the mean rate of source supply. Values are mean ± standard errors, different letters mean that the difference of the means between treatments was significant (LSD test, *P* < 0.05). *F* values under ANOVA analysis were provided. And ns means no significant, *P < 0.05. **P < 0.01. ***P < 0.001.

**Table 4:** Parameter evaluation of foxtail millet sink dynamics under different fertilization treatments.

Year	Treatment	Estimated parameter values of sink						
		W_max,h_	t_b,o_	t_e,o_	t_m,o_	S_max,h_	${\bar{S}}_{h}$	R^2^
		(g m^-2^)	(day)	(day)	(day)	(g m^-2^ day^-1^)	(g m^-2^ day^-1^)	
2022	CT	911.6 ± 25.8 b	27.7 ± 7.6 a	99.5 ± 13.2 a	48.2 ± 8.1 b	18.5	12.8 ± 1.5 ab	0.9867
	N-15	959.3 ± 43.3 ab	20.4 ± 3.2 ab	102.2 ± 13.2 a	59.2 ± 3.3 a	17.4	9.4 ± 1.3 bc	0.9947
	N+15org	953.3 ± 69.0 ab	17.0 ± 4.5 b	89.0 ± 8.2 a	59.7 ± 2.5 a	21.4	10.7 ± 0.4 ab	0.9890
	N-30	885.7 ± 41.8 b	19.4 ± 5.9 ab	100.0 ± 3.5 a	61.2 ± 4.1 a	16.7	11.0 ± 0.4 c	0.9908
	N+30org	1033.9 ± 30.7 a	25.8 ± 1.4 ab	99.8 ± 1.2 a	57.5 ± 0.2 a	20.4	14.0 ± 0.3 a	0.9938
2023	CT	846.6 ± 40.7 b	25.0 ± 7.4 a	98.9 ± 13.0 ab	50.9 ± 9.5 b	16.5	11.5 ± 1.2 ab	0.9870
	N-15	865.2 ± 63.0 b	20.5 ± 4.1 ab	113.7 ± 11.0 a	57.8 ± 3.8 ab	13.4	7.6 ± 1.3 c	0.9886
	N+15org	887.0 ± 35.9 ab	14.7 ± 1.7 b	86.0 ± 1.1 b	61.7 ± 0.5 a	21.9	10.3 ± 0.4 a	0.9895
	N-30	820.1 ± 18.9 b	20.8 ± 6.8 ab	97.0 ± 6.8 b	61.4 ± 4.4 a	16.5	10.8 ± 0.8 bc	0.9944
	N+30org	952.4 ± 26.6 a	26.7 ± 1.3 a	99.9 ± 2.2 ab	56.9 ± 0.2 ab	18.9	13.0 ± 0.5 a	0.9945
Year (Y)		11.2**	Ns	Ns	Ns	\	5.0*	\
Chemical fertilizer reduction (C)		Ns	Ns	Ns	6.2*	\	Ns	\
Organic fertilizer substitution (O)		12.0**	9.5**	4.1*	7.2**	\	Ns	\
Y × C		Ns	Ns	Ns	Ns	\	Ns	\
Y × O		Ns	Ns	Ns	Ns	\	Ns	\

Sink parameter description: W_max,h_, the maximum head biomass; t_b,o_, time point at booting; t_e,o_, timing when the rate of sink demand is decreased to zero; t_m,o_, the moment when the maximum rate of sink demand is achieved; S_max,h_, the maximum rate of sink demand; ${\bar{S}}_{h}$, the mean rate of sink demand. Values are mean ± standard errors, different letters mean that the difference of the means between treatments was significant (LSD test, *P* < 0.05). *F* values under ANOVA analysis were provided. And ns means no significant, *P < 0.05. **P < 0.01. ***P < 0.001.

A significant main effect on the dynamics of foxtail millet source biomass was exerted by chemical fertilizer reduction (*p* < 0.05), whereas no significant effect on sink biomass dynamics (*p* > 0.05). A significant reduction in W_max,p_ and a delay in the time required to reach peak source biomass (t_e,p_) were induced by the decreased chemical fertilizer application rate; specifically, compared with the CT treatment, W_max,p_ was decreased by 6.8–9.2% and 9.5–12.6% under the N-15 and N-30 treatments, respectively, and t_e,p_ was prolonged by approximately 6–18 days under the N-30 treatment. However, no obvious effect on the dynamics of foxtail millet sink biomass was exerted by chemical fertilizer reduction, and no significant differences in W_max,h_, t_b,o_, and t_e,o_ were observed among the N-15, N-30, and CT treatments (*p* > 0.05). The dynamics of foxtail millet source and sink biomass could be effectively regulated by partial chemical fertilizer substitution with organic fertilizer. Compared with the CT treatment, the time required to reach peak source biomass (t_e,p_) was significantly reduced by 2–4 days under the N + 15org and N + 30org treatments; and W_max,p_ was significantly increased by 12.5–13.4% under the N + 30org treatment compared with the CT treatment (*p* < 0.05). Under sink biomass dynamics, W_max,h_ was significantly increased and the time required to reach peak sink activity (t_m,o_) was prolonged by partial chemical fertilizer substitution with organic fertilizer. Compared with the CT treatment, W_max,h_ was increased by 3.2–9.1% (*p* > 0.05) and significantly increased by 9.8–15.7% (*p* < 0.05) under the N + 15org and N + 30org treatments, respectively, while t_m,o_ was prolonged by 10–11 days and 6–9 days, respectively.

### 3.4 Sink and source relationships

**Figure 4:**
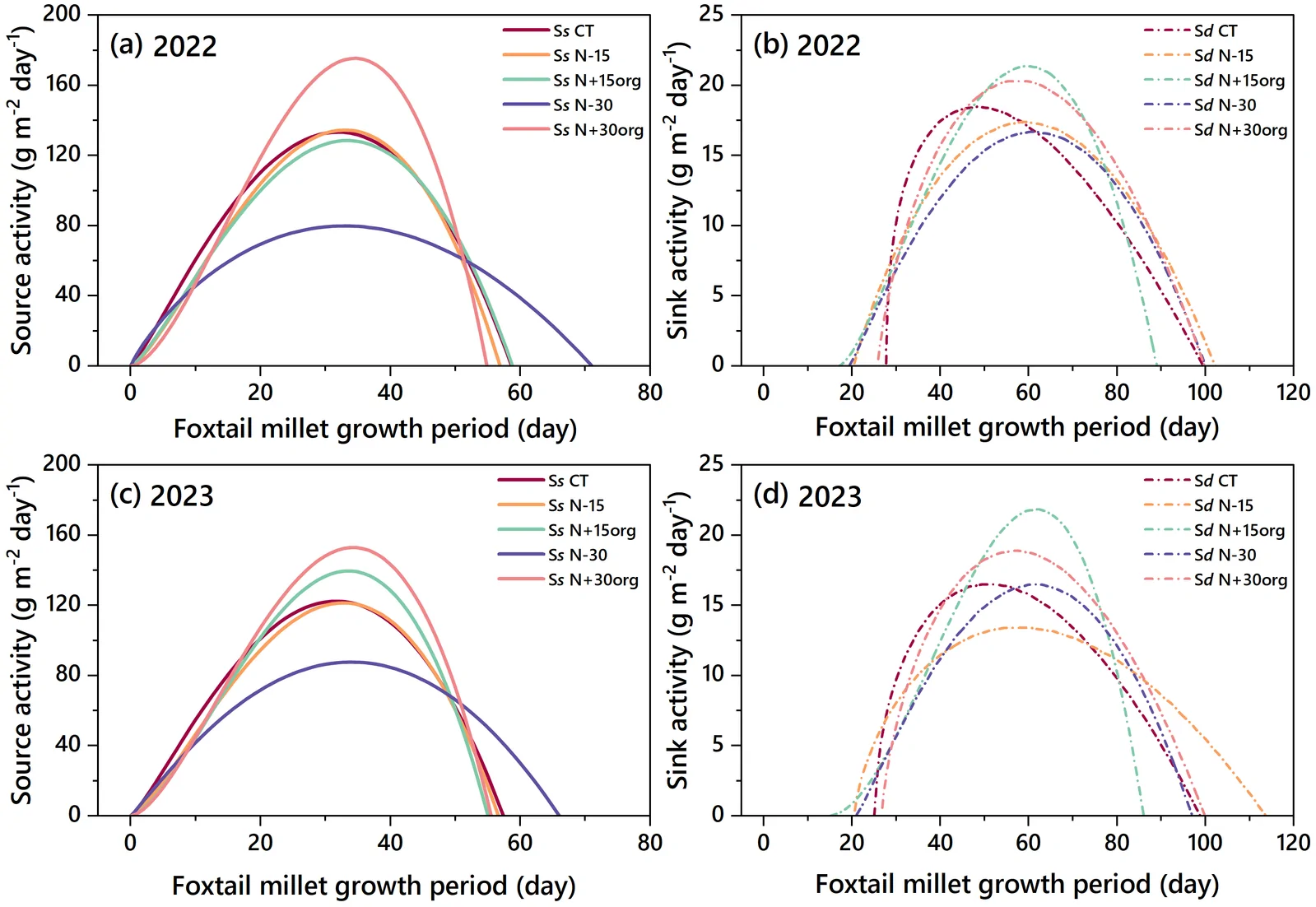
The source-sink activity curve reflects the dynamic changes of source supply and sink demand under different treatments in 2022-2023, and the source-sink curve is calculated by formula (3) and (4), respectively. Panels **(a)** and **(c)** display source activity while **(b)** and **(d)** display sink activity, with each treatment denoted by different colored lines.

The daily variations of foxtail millet source and sink activities, calculated via (Equations 3, 4), are presented in **Figure 4**. A trend of an initial increase followed by a decrease was exhibited by both the daily source and sink activities of foxtail millet; furthermore, both the peak magnitude and the time required to reach maximum activity of the source activity curve were found to be greater than those of the sink activity curve. The maximum source activity (S_max,p_) was found to range from 87.7 to 175.5 g m⁻² day⁻¹, the average source activity ( ${\bar{S}}_{p}$) was determined to be 14.2–21.6 g m⁻² day⁻¹, and the time required to reach the maximum source activity was estimated at approximately 31–34 days. Concurrently, the maximum sink activity (S_max,h_) ranged from 13.4 to 21.9 g m⁻² day⁻¹, the average sink activity ( ${\bar{S}}_{h}$) was measured at 7.6–13.0 g m⁻² day⁻¹, and the time required to reach the maximum sink activity was observed to be approximately 48–61 days.

A significant prolongation of the source supply duration (t_e,p_) and reductions in the average source and sink activities ( ${\bar{S}}_{p}$ and ${\bar{S}}_{h}$) were induced by the 30% N fertilizer reduction (*p* < 0.05), whereas no significant differences in source and sink activities were detected between the 15% N fertilizer reduction treatment (N-15) and the CT treatment (*p* > 0.05). Compared with the CT treatment, an extension of 8–12 days in t_e,p_ was observed under the N-30 treatment, under which ${\bar{S}}_{p}$ and ${\bar{S}}_{h}$ were significantly decreased by 21.9–27.3% and 6.1–14.1%, respectively (*p* < 0.05). The source and sink activities of foxtail millet could be effectively enhanced by the 30% organic fertilizer substitution, under which ${\bar{S}}_{p}$ and ${\bar{S}}_{h}$ were increased by 7.1–9.1% and 9.3–13.0%, respectively, under the N + 30org treatment compared with the CT treatment (*p* < 0.05), while no significant differences in source and sink activities were exhibited under the N + 15org treatment (*p* > 0.05).

The source-sink supply and demand relationships of foxtail millet are reflected by the source supply, sink demand, and sink-to-source ratio (**Table 5**). The highest source supply and sink demand were exhibited by the N + 30org treatment; correspondingly, the lowest source supply and sink demand were observed under the N-30 treatment. Nevertheless, source-sink utilization was significantly enhanced by both N fertilizer reduction (N-15 and N-30) and organic fertilizer substitution (N + 15org and N + 30org); compared with the CT treatment, the sink-to-source ratio was significantly increased by 8.2–14.8% and 8.7–11.4% under the chemical fertilizer reduction and organic fertilizer substitution treatments, respectively (*p* < 0.05).

**Table 5:** Difference characteristics of source supply, sink demand and source-sink ratio of foxtail millet under different fertilization treatments.

Year	Treatment	Sink demand	Source supply	Sink to source ratio
		(g m^-2^)	(g m^-2^)	(%)
2022	CT	911.6 ± 25.8 b	1158.4 ± 24.5 a	78.7 ± 1.8 b
	N-15	959.3 ± 43.3 ab	1060.5 ± 21.7 bc	90.4 ± 2.6 a
	N+15org	953.3 ± 69.0 ab	1085.7 ± 24.6 b	87.7 ± 4.4 a
	N-30	885.7 ± 41.8 b	1020.0 ± 25.1 c	86.8 ± 2.3 a
	N+30org	1033.9 ± 30.7 a	1185.9 ± 20.4 a	87.2 ± 1.5 a
2023	CT	846.6 ± 40.7 b	1043.8 ± 17.3 a	81.1 ± 2.9 b
	N-15	865.2 ± 63.0 b	957.9 ± 26.6 bc	90.3 ± 4.3 a
	N+15org	887.0 ± 35.9 ab	995.1 ± 22.9 b	89.1 ± 1.6 a
	N-30	820.1 ± 18.9 b	934.0 ± 27.0 c	87.8 ± 0.6 a
	N+30org	952.4 ± 26.6 a	1079 ± 18.6 a	88.2 ± 1.0 a
Year (Y)		11.2**	19.0***	Ns
Chemical fertilizer reduction (C)		Ns	40.9***	19.7***
Organic fertilizer substitution (O)		12.0**	28.4***	21.7***
Y × C		Ns	Ns	Ns
Y × O		Ns	Ns	Ns

Values are mean ± standard errors, different letters mean that the difference of the means between treatments was significant (LSD test, *P* < 0.05). *F* values under ANOVA analysis were provided. And Ns means no significant, *P < 0.05. **P < 0.01. ***P < 0.001.

### 3.5 Association between fertilization, yield, quality, photosynthesis, sink, and source relationships

**Figure 5:**
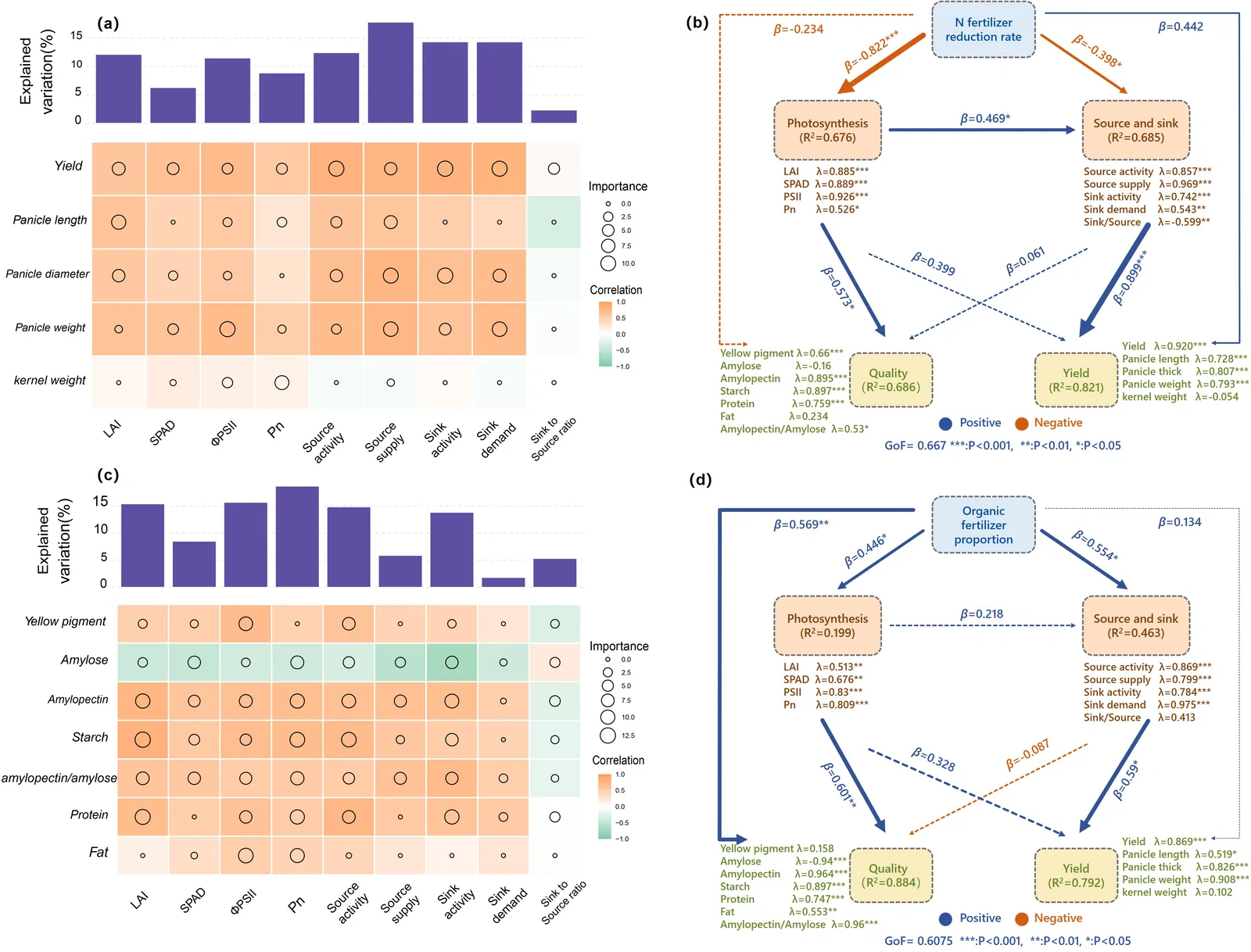
Pearson correlation analysis and random forest importance assessment between different effect factors (Pn, SPAD, LAI, ΦPSII, FV/FM, S_p_, S_h_, source supply, sink demand, sink/source ratio) and foxtail millet yield composition **(a)** and quality characteristics **(c)**. The least squares path model (PLS-PM) constructed the effects of different N fertilizer reduction rates and organic fertilizer substitution rates on the photosynthetic capacity, source-sink relationship, quality characteristics, and yield composition of foxtail millet.

The correlations and importance of foxtail millet canopy characteristics (Pn, SPAD, LAI, ΦPSII, FV/FM), sink, and source relationships (source activity, sink activity, source supply, sink demand, and sink-to-source ratio) in relation to grain quality attributes and yield components are presented in **Figures 5a, c**. Significant positive correlations among canopy characteristics, grain quality attributes, yield components, sink, and source relationships were indicated by Pearson correlation analysis (*p* < 0.05); however, it is noteworthy that no significant correlation was detected between the sink-to-source ratio and grain quality or yield (*p* > 0.05). The relative impacts of different contributing factors on grain yield and quality were reflected, and the primary influencing factors were identified through the importance degrees by Random Forest importance assessment (**Figures 5a, c**). Among the indicators characterizing grain yield and panicle weight were close correlations with sink-source activity and sink-source capacity, with relatively high explained variation being obtained. The grain quality attributes of foxtail millet were closely associated with LAI, ΦPSII, Pn, sink, and source activity. Specifically, the yellow pigment content was synergistically regulated by ΦPSII and source activity, whereas these grain-quality traits had close correlations with LAI, SPAD, ΦPSII, Pn, source, and sink activities.

The causal pathways through which chemical fertilizer reduction and organic fertilizer substitution influenced foxtail millet photosynthetic characteristics, grain quality, yield, sink, and source relationships were reflected by the partial least squares path modeling (PLS-PM), respectively (**Figures 5b, d**). No significant direct relationship was observed between chemical fertilizer reduction and grain quality or yield (*p* > 0.05). However, the significant negative effects of N fertilizer reduction on photosynthetic characteristics, sink, and source relationships of foxtail millet may lead to indirect negative regulation of N fertilizer reduction on quality and yield of foxtail millet (*p* > 0.05). A significant direct positive effect on grain quality (*p* < 0.05) was exerted by organic fertilizer substitution, whereas no significant direct positive effect on grain yield (*p* > 0.05) was detected. An indirect positive regulatory role on grain yield was exhibited by organic fertilizer substitution through its significant positive effect on the sink and source relationships (*p* < 0.05). Significant positive effects of the sink and source relationships on grain yield (*p* < 0.05) were observed in both PLS-PM models, while significant positive effects of photosynthetic characteristics on grain quality (*p* < 0.05) were also detected.

## 4 Discussion

### 4.1 Effects of N fertilizer reduction and organic fertilizer substitution on foxtail millet yield and quality

Under the dual context of global climate change and the rapid intensification of agriculture, the traditional production management concept of applying high amounts of chemical fertilizer, which solely pursues high crop yields, has been shifted toward green, low-carbon, and energy-saving practices (Li et al., 2026). Soil acidification and groundwater eutrophication have been accelerated by long-term excessive application of chemical N fertilizer, whereby the physical and biological structures of soil micro-ecosystems have been disrupted, and the sustainable production capacity of crops in arid and semi-arid regions has been severely constrained (Palmero et al., 2026). Consequently, chemical fertilizer reduction has been accepted as a crucial strategic consensus for sustainable agricultural development, soil health protection, and farmland ecological balance (Hu et al., 2023; Chen et al., 2025). In the present study, the yield characteristics of foxtail millet under different N reduction ratios were explored; it was revealed that grain yield was only slightly decreased by 1.0–5.7% (*p* > 0.05) under a 15% reduction in N fertilizer application. However, when the N fertilizer reduction reached 30%, single panicle weight and grain yield were significantly decreased by 5.6–8.9% and 9.5–11.2% (*p* < 0.05), respectively; concurrently, plant height, stem diameter, panicle length, and panicle diameter were also negatively affected. Although foxtail millet is characterized as a typical crop with prominent barren-resistant adaptability, panicle differentiation and grain formation are still restricted by excessive N fertilizer reduction, leading to weak seedlings, thin stems, and small panicles (Mao et al., 2024). Yield penalties induced by N fertilizer reduction can be effectively mitigated by substituting a portion of chemical fertilizer with organic fertilizer; specifically, grain yield was enhanced by 7.2–14.9% when 15% of chemical N was replaced by organic fertilizer, whereas a significant increase of 9.3–11.0% in single panicle weight and a significant enhancement of 17.3–23.5% in grain yield (*p* < 0.05) were achieved when the organic substitution ratio reached 30%. Currently, an organic fertilizer substitution ratio of approximately 20–50% is considered an appropriate fertilizer combination to enhance foxtail millet productivity and coordinate nutrient supply and demand (Nie et al., 2025). The primary advantage of organic fertilizer in promoting crop growth stems from the systematic improvement and remodeling of soil physical, chemical, and biological properties (Uddin et al., 2025). Total porosity, aeration, and water retention are effectively increased by the input of organic matter, whereby the soil retention and adsorption capacities for nitrate nitrogen, ammonium nitrogen, available phosphorus, and available potassium are enhanced (Lu et al., 2019). More critically, the activities of key metabolic enzymes in the soil, such as sucrase and urease, can be stimulated by organic fertilizer substitution (Xu et al., 2023); concurrently, the carbon and N metabolic networks of the rhizosphere micro-ecology are reconstructed by increasing the diversity and richness of soil bacterial and fungal communities (Xu et al., 2026), thereby laying a favorable belowground nutrient foundation for high crop yields.

Specific responses to N supply levels and organic fertilizer substitution are exhibited by the basic quality characteristics of foxtail millet. The eating quality, nutritional profiles, and processing suitability of foxtail millet are determined by starch and protein contents (Ma et al., 2026). In this study, the amylopectin/amylose ratio in foxtail millet grains was significantly increased by 7.5–17.9%, and the protein content was significantly enhanced by 6.6–12.2% (*p* < 0.05) under organic fertilizer substitution. A stable, slow-release N source can be continuously provided by organic fertilizer substitution to fulfill the late-stage nitrogen requirements for protein synthesis (Wu et al., 2024). Furthermore, carbon metabolism may be enhanced and starch branching enzyme activities optimized by the abundant trace elements and small-molecule organic carbon present in organic fertilizers; consequently, the amylopectin ratio is improved and the starch structure is optimized concurrently with an increase in total protein content (Nie et al., 2025). Notably, the yellow pigment content of foxtail millet grains is predominantly affected by the N supply level. Under the N-15 and N-30 treatments, the yellow pigment content of grains was significantly decreased by 10.4–12.6% and 15.4–30.1%, respectively (*p* < 0.05). As a core indicator determining the appearance quality of foxtail millet, the primary biochemical component of yellow pigment is characterized as carotenoids (Yang et al., 2017). Carotenoids exist abundantly in chloroplasts in the form of pigment-protein complexes, which are classified as photosynthetic accessory pigments that are highly dependent on N (Zhang et al., 2019). The structural integrity of chloroplasts is directly disrupted and the metabolic pathways of the carotenoid synthetic enzyme system are blocked by severe N deficiency, thereby leading to a significant decline in yellow pigment content (Mng’ong’o and Msungu, 2024). In contrast, the protein synthesis function during the late growth stages of foxtail millet is guaranteed by the slow-release nutrient characteristics of organic fertilizer (Wu et al., 2024), which successfully prevents the negative impacts on yellow pigment decline induced by chemical fertilizer reduction.

### 4.2 Dynamic changes in photosynthetic performance of foxtail millet under chemical fertilizer reduction and organic fertilizer substitution

The ultimate yield of sink organs is directly determined by the crop canopy photosynthetic capacity and the export efficiency of photosynthates (Qiang et al., 2025). The synthesis of RuBP carboxylase (Rubisco) and bioenergetic carboxylation proteins in crop photosynthesis is directly regulated by N involvement (Lu et al., 2025). In the present study, no significant effect on the photosynthetic capacity of foxtail millet was exerted by moderate chemical fertilizer reduction (N-15); however, a significant decrease of 8.3–17.7% in the net photosynthetic rate (Pn) of flag leaves was induced by a 30% reduction in N supply (N-30). During the grain-filling stage of foxtail millet, nutrient translocation from source to sink organs can be accelerated by insufficient N supply, thereby resulting in a decrease in flag leaf photosynthetic capacity (Du et al., 2024). According to previous molecular studies, a significant increase in the expression of autophagy-related genes (ATGs) under N deficiency conditions has been demonstrated in contemporary research; furthermore, low N is recognized as one of the direct causes driving the decline in photosynthetic rate (Xing et al., 2024). Conversely, the organic fertilizer substitution strategy was associated with a slower decline in canopy photosynthetic performance after anthesis, thereby maintaining higher physiological activity. Under the condition where 30% of chemical fertilizer was substituted with organic fertilizer (N + 30org), Pn was enhanced by 10.6–21.2%, transpiration rate (Tr) by 12.1–24.4%, and stomatal conductance (Gs) by 9.6–16.4% during the late grain-filling stage (30–40 days after anthesis); concurrently, a 13.2–15.7% increase in the actual photochemical efficiency (ΦPSII) and a 7.0–11.3% increase in the leaf area index (LAI) were also achieved. The increase in leaf area during the late grain-filling stage under the N + 30org treatment implies that organic fertilizer substitution was associated with a slower decline in leaf photosynthetic function, which prolonged the photosynthetic duration and potentially enhanced the canopy light interception efficiency of the foxtail millet population. Concurrently, the increase in actual photochemical quantum efficiency under organic fertilizer substitution reflects that N allocation in key components of the electron transport chain and carboxylation reactions may be optimized by organic fertilizer, whereby the capacities of both light and dark reactions are synergistically enhanced (Qiang et al., 2025). Moreover, in arid and semi-arid agricultural systems, abscisic acid signaling is typically triggered by mild to moderate water stress during the grain-filling stage, by which leaf stomatal closure is induced to reduce transpirational water loss. The proportion of water-stable macroaggregates can be increased by organic fertilizer inputs, as demonstrated in existing literature, through which soil porosity, water-holding, and nutrient-retention capacities are substantially enhanced (Lu et al., 2019; Wu et al., 2024). In the present study, under the rainfed conditions without artificial irrigation, high levels of Gs and Tr were continuously maintained throughout the grain-filling stage under the N + 30org treatment, indicating that a more sufficient and stable water supply could be provided by organic fertilizer for foxtail millet; consequently, unobstructed CO_2_ substrate intake could be maintained by flag leaves while sustaining high stomatal conductance, whereby the stomatal limitation of photosynthesis was effectively alleviated.

### 4.3 Regulatory effects of N fertilizer reduction and organic fertilizer substitution on sink and source relationships of foxtail millet

The final yield formation of foxtail millet is inherently characterized in plant physiology as a physiological and biochemical process in which photosynthates are produced in the “source” (vegetative organs, such as leaves and stems) and subsequently translocated and accumulated in the “sink” (reproductive organs, such as panicles and grains) (Pabuayon et al., 2025). To capture and quantify this extremely complex dynamic developmental characteristic on a temporal scale, mathematical modeling and quantitative analysis of source-sink dynamics throughout the entire growth period of foxtail millet were performed using a sigmoidal beta (β) growth function in the present study (Yin et al., 2009). Compared with traditional Logistic models, Richards functions, or Gompertz equations, the paramount advantage of the beta function lies in the rigorous and easily interpretable biophysical significance possessed by its parameters (Shao et al., 2021). The maximum biomass reserves (W_max,p_ and W_max,h_), time nodes of complete growth termination (t_e,p_ and t_e,o_), maximum absolute growth rates (S_max,p_ and S_max,h_), and time nodes of maximum activity appearance (t_m,p_ and t_m,o_) of the crop can be predicted by this model (Fang et al., 2024; Fei et al., 2024; Liao et al., 2024; Wang et al., 2025b). More importantly, growth rates at the initiation and termination of growth are mathematically allowed to be zero by the beta function model, which ensures a strictly deterministic growth endpoint for annual crops; consequently, a high goodness-of-fit is exhibited during model simulation (He et al., 2025). As an annual C4 cereal crop, foxtail millet exhibits a strictly bounded phenological duration where vegetative and reproductive organ growth completely ceases at physiological maturity. The Beta function mathematically enforces growth rates (dW/dt) to strictly equal zero at both growth initiation and termination. This structural property allows for an accurate, non-biased estimation of source-sink capacity, activity duration, and maximum growth rate, providing a highly reliable quantitative basis for evaluating fertilization responses in foxtail millet. In this experiment, a coefficient of determination (R^2^) of over 98% was achieved when the beta function was applied to fit the temporal variability of foxtail millet source and sink biomass, which fully demonstrates that high accuracy and scientific validity are possessed by this method in quantitatively evaluating the source-sink dynamic characteristics of foxtail millet.

Based on the characteristic parameters derived from the beta function analysis, the regulatory mechanisms of N reduction and organic fertilizer substitution on the source-sink capacity, activity, and utilization efficiency of foxtail millet were revealed. No significant effects on the source-sink dynamic curves and characteristic parameters of foxtail millet were exerted by moderate N reduction; however, under the 30% N reduction condition, the maximum source capacity (W_max,p_), average source activity ( ${\bar{S}}_{p}$), and sink activity ( ${\bar{S}}_{h}$) of foxtail millet were significantly decreased by 9.5–12.6%, 21.9–27.3%, and 6.1–14.1%, respectively (*p* < 0.05). Nevertheless, it is noteworthy that the sink-to-source ratio was significantly increased by 8.2–14.8% under the N fertilizer reduction condition, a phenomenon that is recognized as a typical source-sink coordination strategy exhibited by crops in response to “source limitation” (You et al., 2026). When exogenous N supply is insufficient, the post-anthesis leaf photosynthetic assimilation capacity is decreased, and the source-sink allocation ratio is passively coordinated by foxtail millet, whereby non-structural carbohydrates and soluble proteins stored in vegetative organs are mobilized in advance to translocate to the grains (Lu et al., 2025); consequently, the maximum source capacity (W_max,p_) is forced to decrease, and the time required to reach peak source capacity (t_e,p_) is prolonged by 8–12 days. Under this strategy, the photosynthetic capacity of foxtail millet is maintained only during the early grain-filling stage (**Figure 1**); however, due to the fundamental deficiency in source capacity (W_max,p_), the photosynthetic capacity of foxtail millet is significantly reduced during the late grain-filling stage, which ultimately leads unavoidably to decreases in sink capacity (W_max,h_) and grain yield.

Positive expansion and regulatory effects on the sink and source relationships of foxtail millet were exerted by the chemical N fertilizer substitution with organic fertilizer strategy. Compared with the CT treatment, the average source activity ( ${\bar{S}}_{p}$), sink activity ( ${\bar{S}}_{h}$), maximum source biomass (W_max,p_), maximum sink biomass (W_max,h_), and sink-to-source ratio were all significantly enhanced under the N + 30org treatment. In relevant studies, an increase in source capacity (W_max,p_) and a reduction in the source-sink discrepancy were found by Pan et al. (2022) when 20% of chemical N fertilizer was replaced with organic fertilizer, thereby establishing a more balanced sink and source relationships to enhance rice yield. Similarly, an improvement in sink capacity and grain yield through additional organic fertilizer application was found by Fei et al. (2024) to be primarily driven by the enhancement of source supply and sink growth activity. In the present study, a significant alteration in the growth and development timeline of foxtail millet was additionally observed under organic fertilizer substitution; specifically, the time required to reach peak source biomass (t_e,p_) was shortened by 2–4 days, and the time required to reach peak sink activity (t_m,o_) was prolonged by 6–11 days, through which the effective grain-filling duration of foxtail millet was efficiently extended, allowing photosynthates to be continuously translocated to the grains. In conclusion, prolonged grain filling and increased yield can be achieved by the organic fertilizer substitution strategy through expanding source capacity, boosting source and sink activities, and modulating the sink-to-source ratio.

### 4.4 Key factors and effective pathways for enhancing foxtail millet yield and quality

The final yield and quality of foxtail millet are generally governed by complex soil physicochemical environments and intrinsic plant biochemical metabolism. From the perspective of field management, it has been widely demonstrated by current research that soil nutrient availability and persistence represent the primary factors influencing crop yield and quality, such as soil moisture conservation capacity, basal available nutrient content, rhizosphere microbial activity, and network stability (Wu et al., 2024; Lin et al., 2025). Concurrently, plant internal carbon-nitrogen metabolism, photosynthesis during the grain-filling stage, and the spatiotemporal dynamic changes of sink and source relationships are recognized as the core elements determining crop yield and nutritional quality (Ye et al., 2025). In the present study, based on Random Forest importance assessment and partial least squares path modeling (PLS-PM), the yellow pigment, starch, and protein contents of foxtail millet were found to be predominantly influenced by crop photosynthetic factors, whereas grain yield was primarily affected by source-sink activity and capacity (**Figure 5**). The nutritional quality of foxtail millet is essentially characterized as the synthesis and assembly of specific biochemical substances within grain cells, where photosynthesis serves as the direct driving force for the conversion of carotenoids, sucrose, and amino acids into yellow pigment, starch, and protein (Zhang et al., 2025). The productivity of the source is determined by crop photosynthesis (Fei et al., 2024; Qi et al., 2025), whereas the source-sink capacity and activity within sink and source relationships are identified as the primary factors determining how much dry matter can be accommodated by grains (Pan et al., 2022). If the capacity of the “sink” is insufficient or the translocation activity between “source” and “sink” is low, substances are merely retained in the stems and leaves and fail to be converted into final yield, regardless of how intensive the canopy photosynthesis is (He et al., 2025; Wang et al., 2025b).

**Figure 6:**
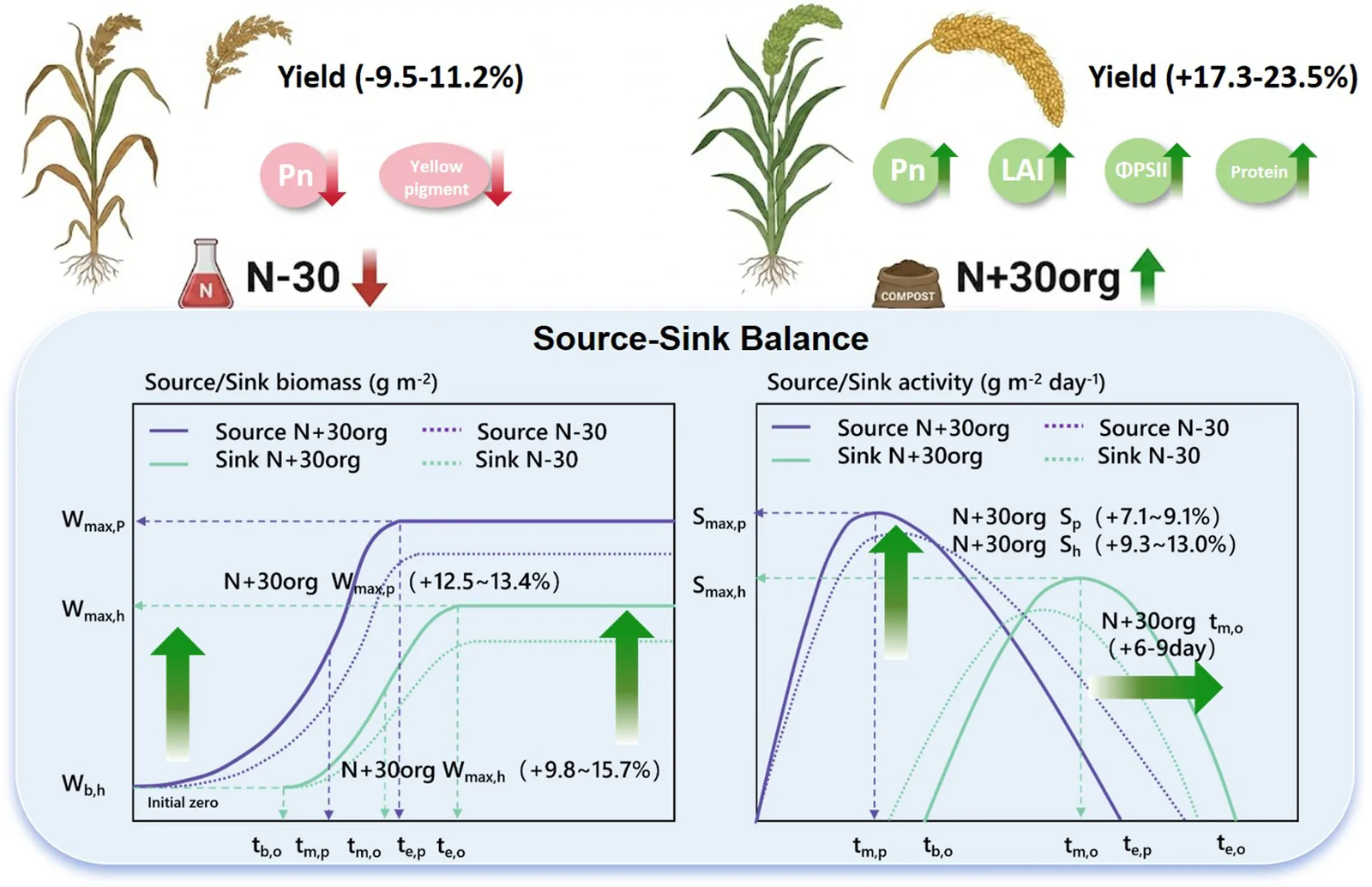
The mechanism diagram showed the changes in agronomic traits and source-sink relationship of foxtail millet under the conditions of 30% nitrogen reduction and 30% organic fertilizer substitution. The proportion calculated in the graph was compared with the conventional fertilization treatment (CT).

The pathways through which chemical N reduction and organic fertilizer substitution influence foxtail millet yield and quality were quantified by the PLS-PM model. No direct relationship was observed between N fertilizer reduction and foxtail millet quality or yield; however, excessive N fertilizer reduction may have a potential indirect negative impact on yield and quality by affecting the photosynthetic characteristics, source, and sink activity of foxtail millet. The feasibility of maintaining stable crop yield under moderate N reduction was demonstrated by our results, under which the source-sink utilization of foxtail millet was improved by reducing unnecessary source organ biomass (decreased W_max,p_) while maintaining photosynthetic rates (Pn and ΦPSII remained unchanged), thereby shifting the sink and source relationships of the plant from inefficient “source-sink consumption” to highly efficient “source-sink utilization”. Conversely, concurrent optimizations of foxtail millet quality and yield can be achieved through the independent regulation of photosynthetic characteristics, sink, and source relationships by organic fertilizer substitution, thereby realizing a transition from stable yield to increased yield under chemical N fertilizer reduction regimes (**Figure 6**). Crop yield potential has been demonstrated in previous studies to be indirectly enhanced by organic fertilizer application from the perspectives of improving soil fertility, ensuring soil health, and ameliorating soil micro-ecological environments (Xu et al., 2023; Uddin et al., 2025; Xu et al., 2026). From the perspective of sink and source relationships, a direct theoretical foundation and a feasible pathway for achieving chemical fertilizer reduction are provided by the present study.

## 5 Conclusion

In the present study, we found that moderate N reduction had no significant effect on grain yield, quality, and source-sink dynamics of foxtail millet. However, the Pn and source activity throughout the entire grain-filling stage were significantly decreased by a 30% reduction in N application, by which the expansion of panicle sink capacity was restricted, ultimately leading to degraded grain quality and reduced grain yield. Conversely, the time required to reach peak sink activity was prolonged, and a slower decline in canopy photosynthetic performance during the mid-to-late grain-filling stages was alleviated by the strategy of substituting 30% of chemical fertilizer with an equivalent amount of organic fertilizer, through which the continuous translocation of assimilates to the panicles was promoted, source-sink capacities were significantly expanded, and source-sink utilization was enhanced. Additionally, foxtail millet yield formation was closely associated with source-sink activity and capacity, whereas grain quality was closely associated with canopy photosynthetic performance. Organic fertilizer substitution was demonstrated in this study to represent an effective pathway for concurrently enhancing foxtail millet yield and quality under chemical fertilizer reduction regimes, thereby providing a theoretical foundation and a practical reference for the coordinated development of foxtail millet quality and quantity.

## Data Availability

The raw data supporting the conclusions of this article will be made available by the authors, without undue reservation.
