# Supplementary material for: Coordinating source-sink dynamics and canopy photosynthesis through organic fertilizer substitution synergistically enhances foxtail millet yield and quality
Source: Front Plant Sci. 2026 Aug 24;17:1938408. doi: 10.3389/fpls.2026.1938408 (PMC13547512; doi:10.3389/fpls.2026.1938408)
Supplement: Supplementary file 1 [file DataSheet1.docx]

**supplementary materials**

Fig. S1. The changes of temperature and rainfall during the growth period of spring foxtail millet from 2022 to 2023.

Fig. S2. (a) Source-sink biomass of foxtail millet during growth period (sink, represented by green line; source, represented by the purple line). (b) Time dynamic changes of source and sink activity. The symbols W_max,p_, W_max,h_, W_b,h_, t_b,o_, t_m,p_, t_m,o_, t_e,p_, t_e,o_, S_max,h_, S_max,p_ are defined in Table S1. In this study, the foxtail millet biomass follows the β S-type growth function, in which the starting point of the source supply and the initial value of the source are assumed to be 0, reflecting the actual situation of the above-ground biomass from zero.

Table S1 List of parameters for source and sink.

| Parameter | | Definition | Unit |
| --- | --- | --- | --- |
| Sink | W_max,h_ | the maximum head biomass | g m^-2^ |
|  | W_b,h_ | the initial head biomass | g m^-2^ |
|  | t_b,o_ | time point at booting | day |
|  | t_e,o_ | timing when the rate of sink demand is decreased to zero | day |
|  | t_m,o_ | the moment when the maximum rate of sink demand is achieved | day |
|  | S_max,h_ | the maximum rate of sink demand | g m^-2^ day^-1^ |
|  | S_h_ | the mean rate of sink demand | g m^-2^ day^-1^ |
| Source | W_max,P_ | the maximum source biomass | g m^-2^ |
|  | t_e,p_ | timing when the rate of source supply is decreased to zero | day |
|  | t_m,p_ | the moment when the maximum rate of source supply is achieved | day |
|  | S_max,p_ | the maximum rate of source supply | g m^-2^ day^-1^ |
|  | S_p_ | the mean rate of source supply | g m^-2^ day^-1^ |
